# The relationship between teacher punishment and student academic achievement: a meta-analysis

**DOI:** 10.3389/fpsyg.2026.1705506

**Published:** 2026-06-23

**Authors:** Shun-yu Li, Jia-hui Zhang, Jia-xin Wei, You-lai Zeng

**Affiliations:** 1 Center for Teacher Education Research, Xinjiang Normal University, Urumqi, China; 2 Institute of Educational Sciences, Huazhong University of Science and Technology, Wuhan, China; 3 Yutian No. 3 Middle School, Tangshan, China; 4 Faculty of Education, Liaoning Normal University, Dalian, China

**Keywords:** meta-analysis, participant characteristics, student academic achievement, study characteristics, teacher punishment

## Abstract

**Background:**

Teachers’ use of “zero tolerance” policies in student discipline has attracted widespread social attention. However, due to the insufficient professional training and guidance, some teachers adopt unscientific punitive methods, such as corporal punishment, suspension, or expulsion, which may be associated with adverse outcomes in students’ academic achievement, cognitive development, and physical and mental health.

**Methods:**

This study conducted a comprehensive meta-analysis of empirical research conducted over the past two decades to examine the relationship between teacher punishment and secondary school student’s academic achievement, with a focus on identifying moderating variables. A distinctive feature of this meta-analysis is its categorization of moderators into participant characteristics, study characteristics, and measurement characteristics, thereby clarifying the multifaceted factors associated with the relationship.

**Results:**

The literature search identified 18 independent effect sizes involving 6,052,011 participants. The heterogeneity test supported the use of a random-effects model. The pooled effect size indicated a weak but statistically significant negative association between teacher punishment and student academic achievement (r = −0.183, 95% CI [−0.221, −0.145], *p* < 0.001). Moderator analyses showed that gender composition, achievement type, and punishment type contributed to variations in the association, whereas grade level and research design did not show significant moderating effects. Publication bias tests did not detect statistically significant evidence of publication bias.

**Conclusion:**

The study found a weak negative association between teacher punishment and student academic achievement. This association was moderated by gender composition, achievement type, and punishment type, but not by grade level or research design. Given the high heterogeneity and the observational nature of the included studies, the findings should be interpreted cautiously as correlational evidence.

## 1 Introduction

Over three decades ago, teachers had already adopted “zero tolerance” policies as a means of disciplining students, a practice that has long drawn widespread societal attention (Kodelja, 2019). For minors who exhibit misconduct or severe behavioral issues at school, the absence of appropriate disciplinary measures would hinder the effective resolution of developmental challenges, including behavior correction and character cultivation. However, due to insufficient professional training and guidance, some teachers fail to adhere to scientific principles and methods when imposing punishment, resorting to inappropriate approaches such as corporal punishment, suspension, or expulsion. Such practices may be associated with adverse outcomes across multiple domains, including academic achievement, cognitive abilities, and physical and mental health. Early research on the effects of teacher punishment on individual academic achievement was pioneered by scholars like Davis and Jordan. Yet, due to variations in research subjects, tools, and measurement methodologies, academic conclusions have been inconsistent and even contradictory. To address this, the present study employs meta-analysis methods to statistically synthesize existing research findings on the relationship between teacher punishment and student academic achievement, aiming to obtain more objective overall effect size. Additionally, it is crucial to explore the influence of objective characteristics of the research subjects (e.g., gender, academic stage) and subjective factors related to research design on this relationship. In the present study, teacher punishment refers to school-based disciplinary practices implemented by teachers or school personnel in response to student misbehavior, including exclusionary punishment, corporal punishment, and general disciplinary punishment when these practices were explicitly linked to academic achievement outcomes.

Moreover, academic achievement has been operationalized in substantially different ways across prior studies, including GPA, course grades, reading achievement, mathematics achievement, and standardized or composite assessments. Similarly, teacher punishment has been measured through different disciplinary practices, such as suspension, expulsion, disciplinary referral, and corporal punishment. Such conceptual and measurement heterogeneity may partly explain the inconsistent findings in the literature and the high between-study heterogeneity in meta-analytic synthesis. Therefore, in addition to gender, grade level, and research design, the present study further coded achievement type and punishment type as moderators to examine whether the association between teacher punishment and student academic achievement varies across different operational definitions.

## 2 Literature review

### 2.1 The concept and measurement of teacher punishment

Before defining teacher punishment in empirical research, it is necessary to clarify that punishment has long been discussed in educational theory as a multidimensional disciplinary practice rather than a single uniform behavior. In the 17th century, John Amos Comenius, hailed as the “father of education”, argued in The Great Didactic that “education should conform to the laws of nature and the nature of children, yet punishment cannot be entirely ruled out”. He further emphasized that those who err should be punished not for their past misdeeds, but to correct their wrongful behavior and prevent recurrence. Education, he maintained, must align with the operations of nature and the inherent patterns of children’s development, never running counter to them—though punishment remains a necessary tool (Keatinge, 1967). John Locke, in Some Thoughts Concerning Education, posited that “good and evil, rewards and punishments are the sole motives of action in rational creatures, and the incentives and restraints by which all mankind are led to work; hence, they ought also be applied to children” (Lockex and Quick, 1880). Johann Friedrich Herbart viewed punishment as a form of student management, asserting that management and education are closely linked, and teaching alone would be futile, especially at the beginning of education are deeply intertwined. Mere teaching, he argued, would prove ineffective—especially in the early stages of education, where instruction alone cannot suffice. At such times, he contended, punitive measures must be employed as a deterrent (Herbart, 2017). Anton Makarenko explicitly put forward the concept of “disciplinary system,” asserting that punishment is both a right and an obligation. A reasonable punitive system, he believed, contributes to forging students’ strong character, cultivate their sense of responsibility, strengthening their willpower and personality, and developing their ability to resist and overcome temptation (Makaranko et al., 1955).

These classical discussions indicate that punishment in education has historically included different meanings, such as moral correction, behavioral management, deterrence, and responsibility cultivation. However, these normative discussions cannot be directly used as operational definitions in empirical meta-analysis. Therefore, the present study focuses on how teacher punishment has been measured in empirical studies and distinguishes among general punishment, exclusionary punishment, and corporal punishment. Straus and Murray defined punishment as the application of physical or psychological actions that may cause pain, aimed at preventing the recurrence of harmful behavior (Straus, 2001). Teacher punishment can be categorized into three types: general punishment, exclusionary punishment, and corporal punishment. General punishment methods include verbal reprimands, revocation of student privileges, and assignment of community services. Exclusionary punishment encompasses disciplinary referrals, in-school suspension, out-of-school suspension, and expulsion (Baker-Henningham et al., 2009). A disciplinary referral involves removing a student from the classroom to meet with administrators, who then determine the consequences. In-school suspension requires the student to remain on campus while facing specified penalties, often accompanied by proactive intervention measures. Out-of-school suspension, modeled after certain American crime control strategies, focuses on isolating “problematic” individuals from those deemed “good” and law-abiding (Umeh et al., 2020). Corporal punishment includes pinching, ear-pulling, hair-pulling, slapping, throwing objects at students, forcing them into painful standing positions, prolonged exposure to sunlight, requiring them to sit in “invisible chairs “(a form of sustained muscle strain), making them hold or carry heavy objects, kneeling on small items like stones, and imposing excessive physical activity without rest (Gershoff, 2017). The primary triggers for teachers to implement exclusionary punishment are student disrespect toward teachers, student interference with teaching activities, and incidents of fighting or bullying. Research indicates that suspension is among the most widely used forms of exclusionary punishment, whereas expulsion is relatively less common (Skiba et al., 2014).

Researchers extracted data on in-school and out-of-school suspensions for a specific academic year from the School Information System (SIS). These records included details for each student, such as the number of suspensions, duration of each suspension, reasons for suspension, and the date of e the incident (Hwang, 2018). The measure students’ perceptions of corporal punishment, researchers use the Student Survey of Corporal Punishment, originally developed by Jacqueline Beekman Bennett. Later, Victor Riley modified Bennett’s instrument to assess elementary school students’ attitude and perceptions toward the use of corporal punishment within the Lancaster School District. The survey comprises ten scenarios, each depicting inappropriate behavior in a school setting. Participants are asked to circle the image corresponding to the punishment they deem appropriate for the described misbehavior. The punishment options include: doing nothing, time-out, loss of privileges, suspension, and corporal punishment. Additionally, a blank space is provided after each set of images for participants to write in an alternative punishment, which they perceive as more appropriate than the listed options. The Student Survey of Corporal Punishment is scored by assigning numerical values (1 to 5) to each response option. By aggregating responses across all scenarios, the survey assesses students’ overall perceptions of punishment: the more frequently participants select corporal punishment as a consequence, the more favorable their attitude toward this discipline method (Smith, 2015).

Because these disciplinary practices differ in severity, mechanism, and potential consequences for students’ learning opportunities, punishment type may influence the magnitude of the association between teacher punishment and academic achievement. For example, exclusionary punishment may reduce achievement by directly removing students from instructional time, whereas corporal punishment may operate through psychological distress, fear, or reduced school engagement. Accordingly, punishment type was coded as an additional moderator in the present meta-analysis to examine whether different disciplinary practices were associated with different effect sizes.

### 2.2 The concept and measurement of academic achievement

Astin once conceptualized academic achievement as the measurable outcomes of both cognitive and non-cognitive factors, specifically the measurable manifestations of intelligence and emotion. He noted that cognitive measurements pertain to behaviors requiring the application of advanced psychological functions such as reasoning and logic, whereas non-cognitive measurements relate to aspects of students’ lives including individual attitudes, aspirations, values, social connections, interpersonal relationships (Astin, 1974). Academic achievement is often measured using students’ academic achievement as the evaluation criterion. This approach is a commonly employed means of assessing individual learning effectiveness, and is regarded as one of the simplest, most effective, and fairest methods for determining academic achievement. A report from The Center for Research and Intervention on School Success (CRIRES) in 2005 states that academic achievement functions as a framework for measuring students’ accomplishments, knowledge, and skills (Davison and Dustova, 2017). This measurement is grounded in the student’s age, prior experiences, and abilities associated with social and educational competencies. To assess academic achievement, educators utilize various types of assessments. Assessment, as a continuous process, yields valuable insights into the learning journey (Davison and Dustova, 2017).

The Wide Range Achievement Test (WRAT) is commonly used to assess school achievement, encompassing tests in reading, spelling, and mathematics. Some studies measure academic achievement using the Measures of Academic Progress (MAP), a computerized adaptive test designed to help schools track students’ academic growth in reading and mathematics. As a standardized assessment, it also supports data-driven decisions regarding student placement and service provision. Another tool employed in research is ACT Aspire, a summative assessment that evaluates student’s growth in English, mathematics, reading, writing, and science from third grade through early high school. Administered via paper-and-pencil or computer formats, ACT Aspire also monitors college and career readiness. Developed by integrating academic research with empirical data, it effectively measures c learning outcomes and provides predictive insights. ACT Aspire uses Performance Level Descriptor (PLD) to illustrate student progress across grade levels, with four categories: (a) Needs support, (b) Approaching, (c) Proficient, and (d) Exceeding (Gohanna, 2017; Tarkington, 2025; Hall et al., 2022). A “Proficient” rating corresponds to scores meeting the ACT readiness benchmark for each grade level (Nelson, 2020). In each wave of the National Longitudinal Study of Adolescent to Adult Health (AddHealth), students report their grades in English, science, social studies, and mathematics. These grades are converted to grade point averages (GPAs) using a standard scale: A = 4.0, B = 3.0, C = 2.0, and D or lower = 1.0 (Duxbury and Haynie, 2020).

These measures do not capture identical aspects of academic achievement. GPA and course grades may reflect students’ overall classroom performance and teacher evaluation, whereas standardized test scores may more directly represent performance on specific cognitive domains such as reading or mathematics. Therefore, differences in the operationalization of academic achievement may contribute to between-study heterogeneity. To address this issue, the present study coded achievement type as a moderator, distinguishing GPA or school grades, subject-specific achievement, and standardized or composite achievement indicators.

### 2.3 The relationship between teacher punishment and student academic achievement

Social control theory aims to reduce antisocial behavior, maintain social order, and enhance the safety and well-being of societies and institutions through the application of discipline. As a tool of social control, discipline can be implemented within groups or communities to achieve internal supervision (informal social control) or externally through the actions of state agents (formal social control). In educational institutions, discipline serves the additional purpose of preparing young people to adapt to the adult roles and responsibilities while ensuring the smooth progression of the learning process. Within the realm of school discipline, researchers often focus on scenarios where social control proves counterproductive. While such counterproductivity may emerge in both overly strict environments (i.e., high-social-control schools) and overly lenient ones (i.e., low-social-control schools), much of the literature on school discipline centers on the former: “In this case, punishment becomes an end itself rather than a means to regulate social order”. For this reason, prior research on school discipline has implicitly drawn on social control theory to trace historical shifts-from overtly racist practices and legacies slavery to modern school disciplinary methods. Recent studies have explicitly applied social control theory to: (a) characterize punitive school environments; (b) examine how students perceive these environments and how such perceptions negatively impact their academic achievement; (c) elaborate on the criminalization of student behavior and its role in widening disciplinary disparities; and (d) explore how disciplinary practices may “collateral consequences” for students not directly targeted by them. Following Perry and Morris, we utilize social control theory to theorize how excessively punitive environments can harm all students (Jabbari and Johnson, 2023).

Current research findings on the relationship between teacher punishment and student academic achievement can be summarized as follows: the first is that exclusionary punishment has been linked to lower levels of student academic achievement. A study analyzed school records of all 6th, 7th, and 8th graders in Texas to examine the extent to which students who received disciplinary actions scored lower in reading and mathematics compared to those who did not. It found statistically significant differences in state assessments results between students subjected to in-school suspension, out-of-school suspension, alternative educational placements, or expulsion and their peers who did not receive such disciplinary measures (Kralevich et al., 2010). Using a nationally representative sample of students, Jabbari and Johnson also found that frequent school suspensions were associated with reduced access to advanced mathematics courses and college enrollment opportunities (Jabbari and Johnson, 2023). Additionally, some studies utilized city- and state-level data to explore the relationship between out-of-school suspensions and academic achievement (in reading and mathematics) among primary, middle, and high school students. Consistent with other research, their findings indicated a significantly negative association between suspensions and student achievement in both subjects (Morris and Perry, 2016; Lacoe and Steinberg, 2018). These studies suggest that exclusionary punishment is negatively associated with academic achievement.

The second type is that high student academic achievement may reduce the likelihood of teacher punishment. One study included data from student and school records of approximately 18,000 K-12 students across 39 schools in a Midwestern school district. It employed multilevel logistic regression and multiple logistic regression to estimate the risk of students receiving one or more suspensions. The results show that students’ achievement in reading and mathematics exams influenced how often teachers used suspension as a disciplinary measure. Specifically, poor academic achievement predicted more frequent teacher punishment (Peguero and Shekarkhar, 2011; Skiba et al., 2014). In other words, higher academic achievement was associated with less teacher intervention in students’ learning and fewer disciplinary actions teacher punishment. However, some studies have drawn opposing conclusion, suggesting that strong academic achievement might also lead to increased teacher punishment. Conversely, a study conducted in five middle and high schools in a medium-sized city in the southeastern United States found that one participant noted no correlation between students’ prior student achievement and teachers’ use of exclusion any punishment (Gregory et al., 2014).

Taken together, prior studies suggest a negative association between teacher punishment and academic achievement, but the direction of this association remains theoretically and empirically complex. Therefore, the present meta-analysis focuses on the magnitude of the association rather than making causal claims about directionality.

### 2.4 The moderating variables of teacher punishment and student academic achievement

#### 2.4.1 Gender

Current research on the gender-based effects of teacher punishment has reached consistent conclusions: most studies confirm that girls are less likely to be suspended than boys, and boys experience significantly more suspensions overall. In terms of gender differences in academic achievement, research has also largely converged: being female is identified as a negative predictor of mathematical performance. Related studies indicate that girls are more likely to pass all exams except those in mathematics and American history. Taylor and Lorimer noted that boys tend to have lower academic achievement in standardized language arts assessments, with boys performing less well than girls in reading and writing (Jordan and Anil, 2009). A study examining the relationship between teacher punishment and student academic achievement uncovered specific gender disparities: sixth-grade boys who were suspended scored higher in mathematics and reading than sixth-grade girls who were suspended. Among students in grades 6–8, girls who received in-school suspensions achieved higher reading scores than their male counterparts. In sixth and seventh grades, boys suspended from school scored higher in mathematics than girls under the same disciplinary measure. Additionally, expelled boys scored slightly lower in reading tests than expelled girls, though this difference was not statistically significant; however, expelled boys had significantly lower average mathematics scores than expelled girls (Kralevich et al., 2010). Contradicting these findings, other studies have suggested that boys subjected to physical punishment are less likely to perform poorly on educational tests compared to girls (Devries et al., 2014). Given these inconsistent results, this study proposes the first hypothesis that gender (male vs. female) may moderate the relationship between teacher punishment and student academic achievement.

#### 2.4.2 Grade

There is no consensus on the stage effect of teacher punishment research, and there is currently limited research across different stages. There is research indicating that there is a significant difference in the frequency of implementing exclusionary discipline among teachers of different grades. The phenomenon of suspension of classes has sharply increased in the middle school stage and continues to rise until the high school grade (Oplatka and Tubin, 2009). In addition, a cross academic study showed that the correlation between the number of suspended students in 6th grade and the number of suspended students in 7th and 8th grades was higher than that in 9th to 12th grades (Raffaele Mendez, 2003). Welsh believes that high school students in alternative schools perform lower than high school students in alternative schools (compared to the region where middle school students are located, mathematics has a standard deviation of −0.86, while high school students have a standard deviation of −0.97) (Welsh, 2022). A meta-analysis on parental corporal punishment shows that corporal punishment has a greater impact on older children than on younger children (Ferguson, 2013). Research on the relationship between teacher punishment and student academic achievement has found that there are differences in academic stages, and most studies suggest that corporal punishment has a significant negative impact on the academic achievement of middle and high school students (Hwang, 2018). In order to further investigate whether academic stage has a moderating effect on the relationship between the two, this study proposes a second research hypothesis that academic stage (middle and high school) may have a moderating effect on the relationship between teacher punishment and student academic achievement.

#### 2.4.3 Research design

In the cross-sectional study design, data are collected at a specific point in time. In contrast, a longitudinal study design involves data collection at predetermined time intervals or in response to predefined events, with continuous tracking of participants. Research indicates that compared to cross-sectional studies, longitudinal design offer unique insights into understanding phenomena such as loneliness prevention. Against this backdrop, the present meta-analysis proposes a third research hypothesis: that study design (cross-sectional vs. longitudinal) may moderate the relationship between teacher punishment and student academic achievement.

#### 2.4.4 Achievement type

Academic achievement has been measured using diverse indicators across empirical studies, including GPA, course grades, reading scores, mathematics scores, and standardized composite assessments. These indicators differ in content coverage, scoring procedures, and sensitivity to school disciplinary practices. For example, exclusionary punishment may have a more direct association with mathematics or reading test scores because it reduces students’ instructional time, whereas GPA may also reflect teachers’ evaluations, classroom behavior, attendance, and school engagement. Therefore, the present study hypothesizes that achievement type may moderate the relationship between teacher punishment and student academic achievement.

#### 2.4.5 Punishment type

Teacher punishment also varies considerably across studies. Some studies focus on exclusionary punishment, such as in-school suspension, out-of-school suspension, expulsion, or alternative school placement, whereas others examine corporal punishment or general disciplinary practices. These forms of punishment may influence academic outcomes through different pathways. Exclusionary punishment may reduce academic achievement by decreasing instructional exposure and weakening school attachment, while corporal punishment may affect learning through emotional distress, fear, or reduced motivation. Therefore, the present study further hypothesizes that punishment type may moderate the association between teacher punishment and student academic achievement.

## 3 Methods

This meta-analysis was conducted in accordance with the PRISMA 2020 guidelines. This meta-analysis was not prospectively registered in PROSPERO or other review registration platforms.

### 3.1 Literature selection criteria

The literature screening process consists of three steps. The first step is retrieval. Since the concept of “teacher punishment” was formally proposed in 2000, the literature retrieval was initiated from that year. Databases including Web of Science, ProQuest, and Google Scholar were searched using the keywords “teacher punishment” and “academic achievement,” with the search deadline set as December 1, 2023. The search strategy combined terms related to teacher punishment and academic achievement using Boolean operators. Search terms included “teacher punishment,” “school discipline,” “corporal punishment,” “suspension,” “expulsion,” “disciplinary referral,” “academic achievement,” “GPA,” “test score,” “reading achievement,” and “mathematics achievement.” Searches were conducted in the title, abstract, and keyword fields when database functions allowed. For Google Scholar, records sorted by relevance were screened until no additional eligible studies were identified. Based on the research topic, 423 articles were initially collected, and 353 of them were excluded in the preliminary screening. The second step was screening, during which an additional 52 articles were excluded according to the screening criteria. The third step was confirmation, in which the full texts of the remaining articles were re-examined, and finally 18 articles were confirmed for inclusion in the meta-analysis.

### 3.2 Inclusion criteria

**Figure 1:**
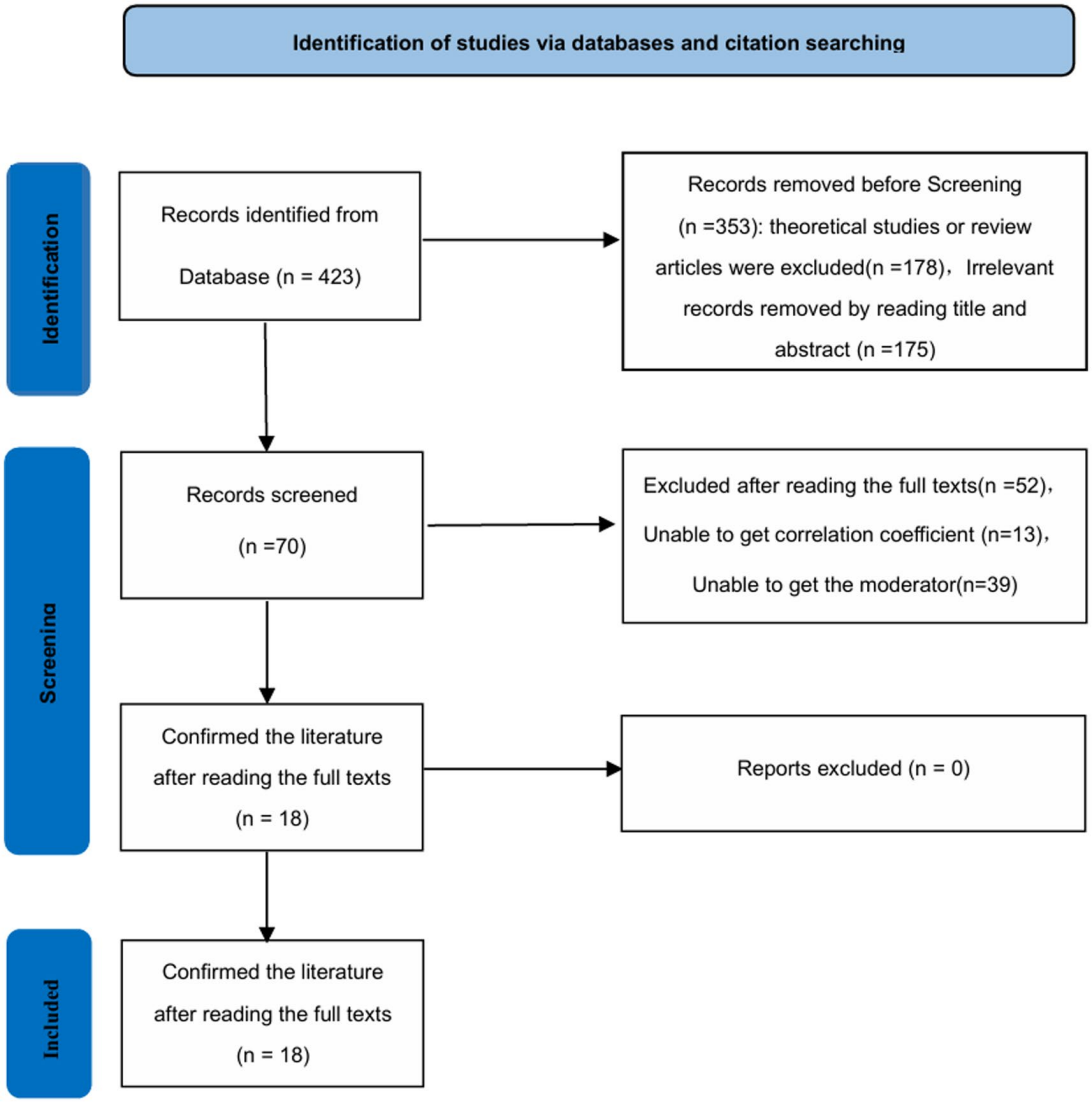
The PRISMA flow chart used to identify studies for detailed analysis of teacher punishment and student academic achievement.

The literature search adhered to four inclusion criteria. First, empirical studies focusing on the relationship between teacher punishment and student academic achievement were included, with purely theoretical or review articles excluded. Second, studies employing quantitative empirical methods were selected, with requirements for complete and clear data—specifically, either reporting the correlation coefficient *r* between teacher punishment and student academic achievement or providing statistical measures (e.g., *F* value, *t* value, or χ^2^ value) that could be converted to *r*. Third, included studies had to explicitly document the measurement tools used to assess both teacher punishment and student academic achievement. Fourth, duplicate articles were excluded, with only one instance retained for studies that reused the same dataset. In total, 18 articles were included in the meta-analysis, containing 18 effect sizes (Figure 1).

### 3.3 Document coding

The articles included in the meta-analysis were coded according to the following categories: (a) reference information, including first author and year of publication; (b) sample size; (c) effect size, namely the correlation coefficient between teacher punishment and student academic achievement; (d) gender composition, represented by the percentage of male participants; (e) grade level, coded as middle school, high school, or secondary/mixed secondary school; (f) study design, coded as cross-sectional or longitudinal; (g) achievement type, coded as standardized or subject-specific achievement, GPA/school grades, standardized/composite achievement, or school-level/unclear achievement; and (h) punishment type, coded as suspension, broader exclusionary punishment, or general/mixed disciplinary punishment.

Before formal coding, two coders jointly coded three pilot studies to clarify the coding rules and resolve potential ambiguities. Two authors then independently coded all eligible studies. Inter-rater agreement was assessed using Cohen’s kappa for categorical variables. The agreement was satisfactory, with Cohen’s *κ* values of 0.91 for grade level, 0.94 for study design, 0.86 for achievement type, and 0.83 for punishment type. For continuous variables, including sample size, correlation coefficient, and male percentage, disagreements were checked directly against the original studies and resolved through discussion. Any remaining disagreements were discussed with a third author until consensus was reached. The final coding results for the 18 target articles are presented in Table 1.

**Table 1:** Coding information of the studies included in the meta-analysis.

Study	Sample size	r	Male%	Grade	Study design	Achievement type	Punishment type
Anderson et al. (2019)	950,745	−0.103	51.30	1	1	1	2
Lehmann and Meldrum (2021)	54,611	−0.19	50.00	2	1	2	1
Ibrahim and Johnson (2020)	16,197	−0.303	53.00	2	2	1	1
Jabbari and Johnson (2023)	25,206	−0.34	50.00	2	2	1	1
Perry and Morris (2014)	16,897	−0.007	50.90	3	1	1	1
Morris and Perry (2016)	16,248	−0.015	50.09	3	1	1	1
Peguero and Shekarkhar (2011)	7,250	−0.124	49.00	2	1	3	3
Kralevich et al. (2010)	998,207	−0.164	49.32	1	1	1	2
Skiba et al. (2014)	43,320	−0.579	68.60	2	1	4	2
Kim (2021)	15,421	−0.093	47.00	3	1	2	2
Rodriguez and Welsh (2022)	3,806,386	−0.199	51.38	3	2	1	2
Welsh (2022)	5,478	−0.685	70.00	3	1	1	2
Duxbury and Haynie (2020)	1909	−0.07	50.69	2	1	2	1
Kinsler (2009)	40,703	−0.088	50.24	1	1	1	1
Hwang (2018)	15,928	−0.015	50.00	3	2	1	1
Jabbari and Johnson (2020)	25,206	−0.05	49.83	2	1	1	1
Gregory et al. (2014)	979	−0.011	50.00	3	1	4	3
Peguero et al. (2015)	11,320	−0.016	49.00	2	1	3	3

Grade was coded as follows: 1 = middle school; 2 = high school; 3 = secondary/mixed secondary school. Study design was coded as follows: 1 = cross-sectional study; 2 = longitudinal study. Achievement type was coded as follows: 1 = standardized or subject-specific achievement; 2 = GPA/school grades; 3 = standardized/composite achievement; 4 = school-level or unclear achievement. Punishment type was coded as follows: 1 = suspension; 2 = broader exclusionary punishment; 3 = general/mixed disciplinary punishment. Broader exclusionary punishment refers to disciplinary practices including in-school suspension, out-of-school suspension, expulsion, alternative educational placement, disciplinary referral, or combined exclusionary disciplinary indicators.

### 3.4 Quality assessment and risk of bias

To evaluate the methodological quality of the included studies, a risk-of-bias assessment was conducted for all 18 studies. Because the included studies were observational rather than randomized intervention studies, the assessment focused on the following domains: sample representativeness, clarity of punishment measurement, clarity of academic achievement measurement, control of potential confounders, appropriateness of study design, and completeness of reported statistical information. Each domain was rated as low risk, moderate risk, high risk, or unclear risk. The overall risk of bias for each study was determined based on the combined assessment across these domains. The results are reported in Table 2.

**Table 2:** Risk-of-bias assessment of included studies.

Study	Samp. rep.	Pun. meas.	Ach. meas.	Conf. ctrl	Design	Stat. rep.	Overall
Anderson et al. (2019)	L	L	L	M	M	L	M
Lehmann and Meldrum (2021)	L	L	L	M	M	L	M
Ibrahim and Johnson (2020)	M	L	L	M	L	L	M
Jabbari and Johnson (2023)	L	L	L	M	L	L	M
Perry and Morris (2014)	L	L	L	M	M	L	M
Morris and Perry (2016)	L	L	L	M	M	L	M
Peguero and Shekarkhar (2011)	M	M	L	M	M	L	M
Kralevich et al. (2010)	L	L	L	M	M	M	M
Skiba et al. (2014)	L	L	M	M	M	L	M
Kim (2021)	M	M	L	M	M	L	M
Rodriguez and Welsh (2022)	L	L	L	M	L	L	M
Welsh (2022)	M	L	L	M	M	L	M
Duxbury and Haynie (2020)	M	L	L	M	M	L	M
Kinsler (2009)	M	L	L	M	M	M	M
Hwang (2018)	L	L	L	M	L	L	M
Jabbari and Johnson (2020)	L	L	L	M	M	L	M
Gregory et al. (2014)	M	M	M	M	M	L	M
Peguero et al. (2015)	M	M	L	M	M	L	M

Samp. rep. = sample representativeness; Pun. meas. = punishment measurement; Ach. meas. = achievement measurement; Conf. ctrl = confounder control; Stat. rep. = statistical reporting. L = low risk; M = moderate risk; H = high risk; U = unclear risk. The overall risk of bias was determined based on the combined assessment across the six domains.

### 3.5 Data analysis

Comprehensive Meta-Analysis 3.0 (CMA 3.0) was used to conduct the meta-analysis, with Pearson’s correlation coefficient r serving as the effect size. Because the included studies differed in sample characteristics, punishment measures, achievement measures, and study design, the random-effects model was used as the primary model. Heterogeneity was evaluated using Cochran’s Q, I^2^*, and* tau^2^ statistics.

Moderator analyses were conducted to explore potential sources of heterogeneity. Categorical moderators, including grade level, study design, achievement type, and punishment type, were examined using subgroup analyses under a mixed-effects model. The percentage of male participants was treated as a continuous moderator and examined using meta-regression. In addition, male percentage was grouped for descriptive subgroup comparison.

Publication bias was assessed using funnel plots, Begg and Mazumdar’s rank correlation test, Egger’s regression test, and the trim-and-fill method. Because funnel plots may involve subjective visual interpretation, the statistical tests and trim-and-fill results were used to provide additional evidence. Sensitivity analysis was conducted using the leave-one-out method, in which one study was removed at a time to examine whether the pooled effect size was disproportionately influenced by any single study.

## 4 Results

### 4.1 Heterogeneity test

Higgins’ I^2^ statistic classifies heterogeneity into low (25%), moderate (50%), and high (75%) based on these thresholds. The heterogeneity test results showed a significant Q-statistic (*p* < 0.001) and an I^2^ value of 99.928%, indicating that the observed variability in the relationship between teacher punishment and student academic achievement stems from true differences in this association rather than sampling error. The between-study variance was substantial (tau = 0.084; tau^2^ = 0.007), suggesting that the between-study variance is sufficient for weight calculation in the meta-analysis. Given the high heterogeneity among effect sizes, a random-effects model is typically employed for meta-analysis—consistent with our earlier inference that the relationship between teacher punishment and student academic achievement is moderated by certain variables. The results are presented in Table 3.

**Table 3:** Results of the heterogeneity test for the effect sizes of teacher punishment and student academic achievement.

Model	Number studies	r	95% interval		Heterogeneity			
			Lower limit	Upper limit	χ^2^	df	*p*	I^2^
FEM	18	−0.179	−0.180	−0.179	23669.784	17	0.000	99.928%
REM	18	−0.183	−0.221	−0.145				

The extremely narrow confidence interval under the fixed-effect model is likely attributable to the very large sample sizes in several included studies and should not be interpreted as evidence of homogeneity. Therefore, the random-effects model was used as the primary model.

The very high I^2^ value indicates substantial between-study heterogeneity. Therefore, the pooled effect size should be interpreted cautiously as an average association across heterogeneous studies rather than as a single uniform population effect. This level of heterogeneity also justifies further moderator analyses. In particular, differences in the operationalization of academic achievement and teacher punishment were examined as additional moderators to determine whether measurement-related factors contributed to the observed heterogeneity.

#### 4.1.1 Assessment of publication bias

**Figure 2:**
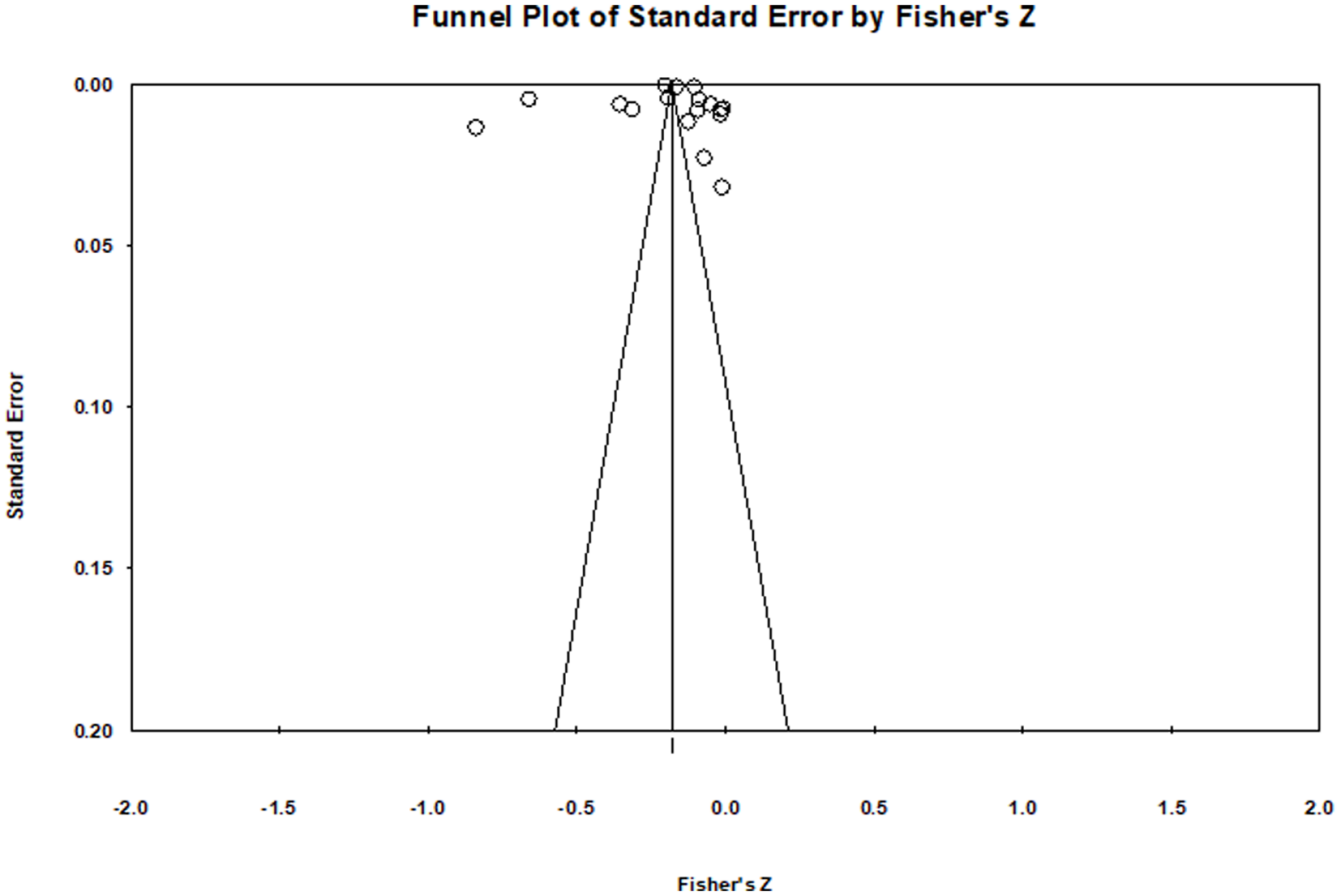
Funnel plot of effect sizes of the correlation between teacher punishment and student academic achievement.

Publication bias was first examined using the funnel plot (Figure 2). Although the included studies were generally distributed around the pooled effect, visual interpretation of funnel plots can be subjective, especially when the number of studies is relatively small.

Begg and Mazumdar’s rank correlation test, Egger’s regression test, and the trim-and-fill method were further used to evaluate publication bias. The results are presented in Table 4.

**Table 4:** Publication bias assessment.

Method	Statistic	*p*	Interpretation
Begg and Mazumdar test	Kendall’s tau = 0.230	0.184	No significant evidence
Egger’s regression test	Intercept = 0.371	0.973	No significant small-study effect
Trim-and-fill	Filled studies = 0; Adjusted r = −0.183	—	Pooled effect remained stable

**Figure 3:**
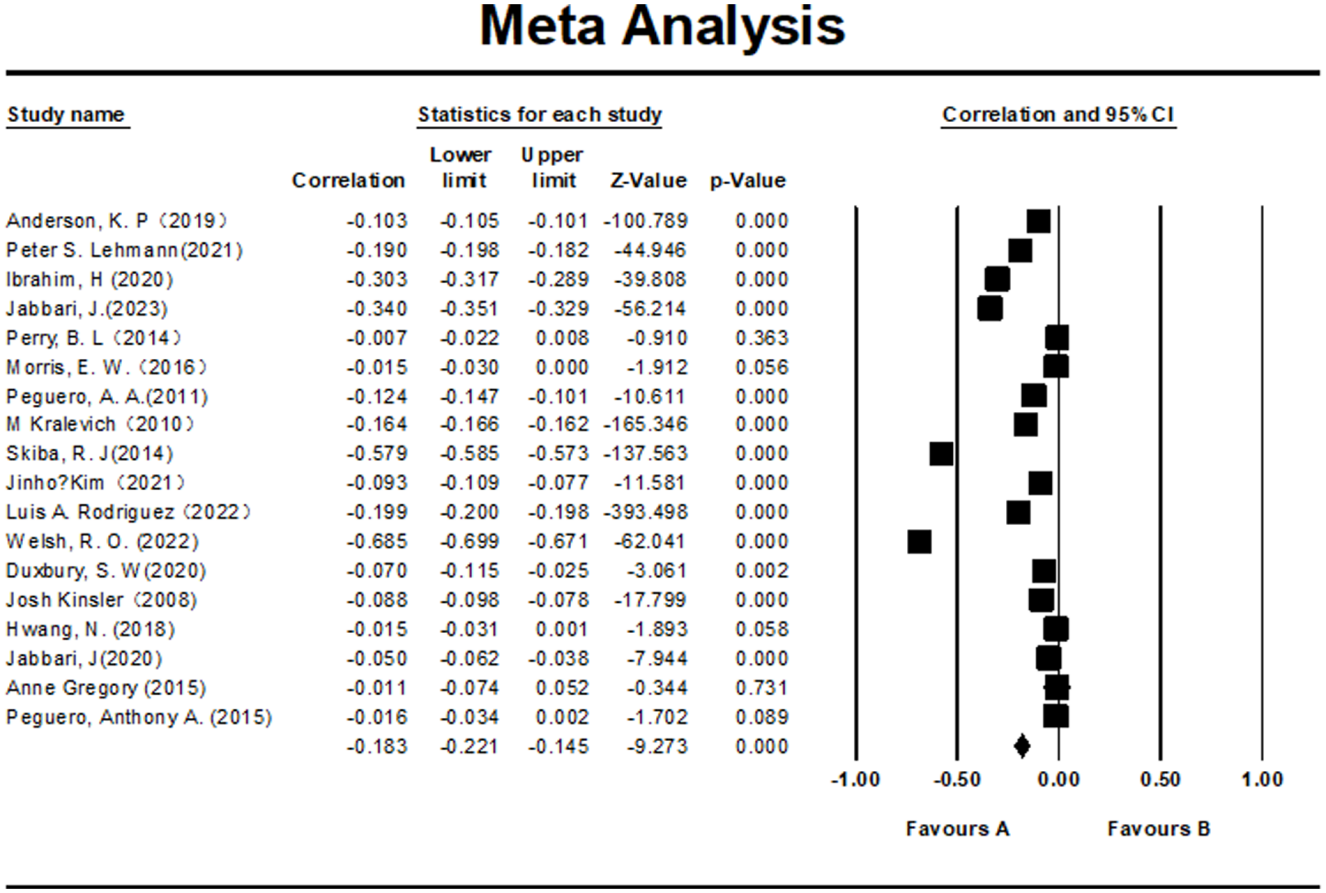
Forest plot of the association between teacher punishment and student academic achievement.

Begg and Mazumdar’s rank correlation test did not indicate significant publication bias (Kendall’s tau = 0.230, *p* = 0.184). Egger’s regression test also did not detect significant small-study effects (intercept = 0.371, *p* = 0.973). The trim-and-fill analysis imputed no missing studies, and the adjusted pooled effect remained unchanged (adjusted r = −0.183). These results did not provide statistically significant evidence of publication bias, although the relatively small number of included studies means that publication bias tests should still be interpreted cautiously (see Figure 3).

#### 4.1.2 Main effect test

A random-effects model was applied to test the main effect using the eligible studies. This meta-analysis included 18 independent samples, with a total of 6,052,011 participants. Results from the random-effects model showed a correlation coefficient of −0.183 between teacher punishment and student academic achievement (95% CI = [−0.221, −0.145], Z = -9.273, *p* < 0.001), indicating a weak negative association between the two variables. Given that most included studies were observational, the pooled effect should be interpreted as an association rather than causal evidence.

#### 4.1.3 Moderating effect test

Moderator analyses were conducted to examine whether the association between teacher punishment and student academic achievement varied by participant characteristics, study characteristics, and measurement characteristics. Grade level, study design, achievement type, and punishment type were treated as categorical moderators and examined using subgroup analyses under a mixed-effects model. The percentage of male participants was treated as a continuous moderator and examined using meta-regression; it was also grouped for descriptive subgroup comparison. The results are presented in Table 5.

**Table 5:** Moderator analyses of the relationship between teacher punishment and student academic achievement.

Moderator	Category		k	r	95% CI	Qb (df)	*p*
Participant characteristics	Male%	39–51%	13	−0.093	−0.134, −0.053	134.166(2)	0.000
		51–61%	3	−0.203	−0.281, −0.121		
		more than 61%	2	−0.634	−0.692, −0.568		
Study characteristics	Grade	middle school	3	−0.118	−0.236, 0.003	2.294(2)	0.318
		high school	8	−0.220	−0.290, −0.148		
		secondary school	7	−0.168	−0.245, −0.089		
	Study design	cross-sectional study	14	−0.173	−0.231, −0.114	0.501(1)	0.469
		longitudinal study	4	−0.217	−0.322, −0.108		
Measurement characteristics	Achievement type	standardized or subject-specific achievement	11	−0.191	−0.228, −0.154	13.419(3)	0.004
		GPA/school grades	3	−0.120	−0.199, −0.039		
		standardized/composite achievement	2	−0.070	−0.175, 0.036		
		school-level or unclear achievement	2	−0.325	−0.750, 0.291		
	Punishment type	suspension	9	−0.122	−0.203, −0.040	32.674(2)	0.000
		broader exclusionary punishment	6	−0.329	−0.386, −0.270		
		general/mixed disciplinary punishment	3	−0.052	−0.135, 0.031		

The results showed that gender composition significantly moderated the association between teacher punishment and student academic achievement. Specifically, the negative association was weakest when the proportion of male participants was relatively low or balanced, and it became stronger as the proportion of male participants increased. The subgroup analysis of male percentage showed a significant between-group difference (Qb = 134.166, *p* < 0.001). In contrast, grade level did not show a significant moderating effect (Qb = 2.294, *p* = 0.318), nor did study design (Qb = 0.501, *p* = 0.469).

In response to the high heterogeneity and the conceptual variability in academic achievement measures, achievement type was further examined as an exploratory moderator. The results indicated that achievement type significantly moderated the association between teacher punishment and student academic achievement (Qb = 13.419, *p* = 0.004), suggesting that studies using different academic achievement indicators did not yield identical effect sizes. Punishment type was also examined as an exploratory moderator, and the results showed a significant moderating effect (Qb = 32.674, *p* < 0.001). These findings suggest that differences in the operationalization of both academic achievement and teacher punishment may partly account for the high between-study heterogeneity.

**Figure 4:**
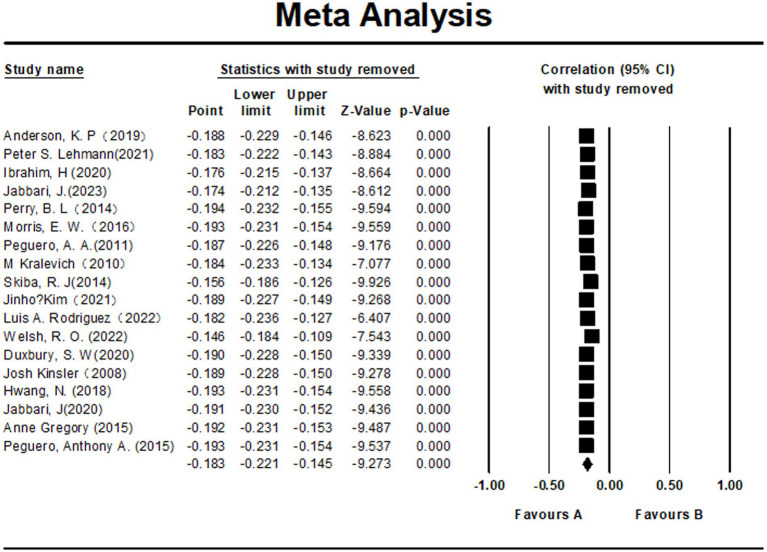
Forest plot of the association between teacher punishment and student academic achievement with one study removed.

Sensitivity analysis was conducted using the leave-one-out method. As shown in Figure 4, the pooled correlation remained negative after sequentially excluding each individual study, with point estimates ranging from −0.194 to −0.146. The corresponding 95% confidence intervals did not cross zero, and the statistical significance of the pooled effect remained unchanged. These results indicate that the overall direction of the association between teacher punishment and student academic achievement was not driven by any single study. However, given the very high heterogeneity, the sensitivity analysis should be interpreted as evidence of directional stability rather than evidence that all studies estimate the same underlying effect.

## 5 Discussion

### 5.1 The relationship between teacher punishment and student academic achievement

This study conducted a meta-analysis of an empirical study on the relationship between teacher punishment and student academic achievement over the past 20 years, encompassing 18 studies with a total of 6,052,011 participants. The results indicate a significant negative correlation between teacher punishment and student academic achievement (r = −0.183, *p* < 0.001), though the correlation strength is weak. Specifically, teacher punishment was negatively associated with students’ academic achievement. This association was observed across studies using different academic outcomes, including reading, mathematics, science-related achievement, GPA, and standardized assessments. However, because the included studies were predominantly observational, this finding should not be interpreted as direct causal evidence. Conversely, some studies suggest that academic achievement may also be associated with the likelihood of receiving teacher punishment: higher academic achievement is generally linked to fewer disciplinary actions. For example, Morris and colleagues used large -scale longitudinal school records to examine the association between suspension and reading and math performance, concluding that exclusionary punishment was associated with lower progress in these subjects (Morris and Perry, 2016). Similarly, research has identified a negative correlation between children’s academic achievement and teacher corporal punishment, suggesting this association may be mediated by emotional functioning—adolescent experiences of corporal punishment are linked to increased anxiety and depression, as well as reduced attention and concentration. This, in turn, may be associated with poorer academic performance, potentially triggering a cycle of more frequent corporal punishment (Baker-Henningham et al., 2009). Additionally, a longitudinal study of U.S. high school students found that school suspensions were negatively correlated with students’ mathematics scores (Jabbari and Johnson, 2023). Unlike previous meta-analyses, this study categorizes moderating variables into the objective characteristics of participants and the subjective characteristics of researchers, clarifying factors that influence the relationship between teacher punishment and academic achievement. The weak negative correlation observed may be explained by the psychological and social stigma associated with punitive measures such as suspension or corporal punishment, which can induce anger, apathy, or detachment in students (Ibrahim and Johnson, 2020). These responses widen academic, social, and emotional gaps between students and their schools, which may be linked to lower academic performance (Oplatka and Tubin, 2009). These findings suggest that schools and teachers should use punitive disciplinary practices cautiously and consider alternative disciplinary strategies that reduce exclusion from learning opportunities and support students’ academic engagement (Oplatka and Tubin, 2009; Ahmad et al., 2013).

### 5.2 Moderating effects and sources of heterogeneity

The moderator analyses suggest that the association between teacher punishment and student academic achievement is not uniform across all studies. In addition to gender composition, measurement-related factors also contributed to variation in effect sizes. In particular, achievement type significantly moderated the association, indicating that GPA, subject-specific achievement, and standardized or composite assessments may capture different dimensions of academic performance. Punishment type also showed a moderating effect, suggesting that exclusionary punishment, corporal punishment, and mixed disciplinary practices may not have equivalent associations with academic achievement.

These findings help explain the extremely high heterogeneity observed in the meta-analysis. Rather than assuming that all included studies measured an identical construct, the results indicate that the pooled effect should be interpreted as an average association across studies with different operational definitions of both teacher punishment and academic achievement.

### 5.3 The moderating role of achievement type

The significant moderating effect of achievement type suggests that different academic indicators may not be equally sensitive to teacher punishment. GPA and school grades reflect not only students’ academic knowledge but also classroom behavior, attendance, teacher evaluation, and school engagement. In contrast, subject-specific test scores, such as reading or mathematics achievement, may more directly reflect learning opportunities and instructional exposure. Standardized or composite assessments may further differ in scoring procedures, content coverage, and comparability across schools. Therefore, the heterogeneity in achievement measures should be regarded as an important methodological source of variation in this field. Future research should report results separately for different academic domains whenever possible, rather than treating academic achievement as a single homogeneous construct.

### 5.4 The moderating role of punishment type

Punishment type also moderated the association between teacher punishment and academic achievement. This finding is theoretically plausible because different forms of punishment may operate through different mechanisms. Exclusionary punishment, such as suspension or expulsion, may influence academic achievement by reducing students’ instructional time, limiting access to academic support, and weakening school attachment. Corporal or general disciplinary punishment may be more closely related to emotional distress, fear, reduced motivation, or negative teacher-student relationships. Therefore, future studies should distinguish among different forms of disciplinary practice and avoid treating all punitive practices as equivalent.

### 5.5 The moderating role of gender

Because the subgroup with more than 61% male participants included only two studies, this result should be interpreted cautiously. The moderating effect of gender composition indicates that the negative association between teacher punishment and academic achievement became stronger in samples with a higher proportion of male students. One possible explanation is that boys are more frequently exposed to disciplinary actions in many school contexts, particularly exclusionary punishment. Greater exposure to disciplinary sanctions may reduce instructional time, weaken school engagement, and increase the likelihood of academic difficulties. However, this interpretation should be treated cautiously because gender differences may be confounded with race, socioeconomic status, school climate, and disciplinary policy. Future studies should use individual-level data to examine whether the observed gender pattern remains after controlling for these factors.

### 5.6 The moderating role of grade

The subgroup analysis showed that grade level did not significantly moderate the association between teacher punishment and student academic achievement. This finding differs from previous research suggesting that the association between school punishment and academic outcomes may vary across grade levels, with stronger negative associations observed in higher grades (Sullivan et al., 2013). One possible explanation is that several included studies used broad grade categories or mixed secondary-school samples, which may have reduced the sensitivity of subgroup comparisons. A meta-analysis on the relationship between school corporal punishment and student academic achievement shows that there is no moderating effect of student stage on these two factors, which is consistent with the results of this study (Visser et al., 2022).

### 5.7 The moderating role of research design

The subgroup analysis showed that study design did not significantly moderate the association between teacher punishment and student academic achievement. This finding suggests that, in the present sample, the pooled association did not differ significantly between cross-sectional and longitudinal studies. However, this result should be interpreted cautiously because the number of longitudinal studies was relatively small. Moreover, study design alone cannot fully address causal inference, especially when punishment and achievement may influence each other over time. Future longitudinal, quasi-experimental, and cross-lagged studies are needed to clarify the temporal ordering and potential reciprocal mechanisms linking teacher punishment and academic achievement.

## 6 Conclusions and limitations

Using meta-analytic methods and Comprehensive Meta-Analysis 3.0 (CMA 3.0) software, this study quantitatively synthesized 18 international studies on teacher punishment and student academic achievement. It objectively estimated the overall effect of their relationship and examined the moderating role of key research characteristics. Results indicate a weak negative correlation between teacher punishment and student academic achievement. This relationship is moderated by gender composition, achievement type, and punishment type, but not by educational stage or research design.

Several limitations should be noted. First, the included studies operationalized academic achievement using different indicators, including GPA, subject-specific test scores, and standardized or composite assessments. Although achievement type was added as an exploratory moderator, the limited number of studies in some categories restricts the strength of the conclusions. Second, teacher punishment was also measured heterogeneously across studies, including suspension, expulsion, corporal punishment, and mixed disciplinary practices. These differences may contribute to the high heterogeneity observed in the meta-analysis. Third, most included studies were observational, and therefore the pooled association should not be interpreted as definitive causal evidence. Fourth, the number of studies was relatively small for some subgroup analyses, especially longitudinal designs and certain punishment types. Finally, although publication bias and sensitivity analyses supported the robustness of the main conclusion, the extremely high heterogeneity indicates that the pooled effect size should be interpreted cautiously.

## 7 Future directions

The study holds significance in three key respects: Theoretically, it quantifies the weak negative correlation between teacher punishment and student academic achievement, thereby enhancing understanding of how disciplinary practices are related to students’ academic outcomes and highlighting the role of gender differences. Practically, it suggests that punitive disciplinary practices should be used cautiously, especially given potential gender-based variations in student responses, and it provides implications for optimizing teaching strategies and educational policy. Methodologically, it identifies limitations in sample distribution, achievement measurement, and punishment measurement, offering guidance for future studies to expand sample coverage, incorporate more longitudinal analyses, and conduct cross-cultural comparisons.

Future research should further distinguish the directionality between teacher punishment and academic achievement. Low achievement may increase the probability of being punished, while punishment may also reduce later achievement by decreasing instructional time and school engagement. Therefore, longitudinal, quasi-experimental, and cross-lagged designs are needed to clarify reciprocal relationships. In addition, future meta-analyses should examine whether punishment-achievement associations vary by cultural context, school policy environment, socioeconomic status, race/ethnicity, school climate, achievement type, and punishment type when sufficient studies become available.

## Data Availability

The original contributions presented in the study are included in the article/supplementary material, further inquiries can be directed to the corresponding author.

## References

[ref1] Ahmad I. Said H. Khan F. (2013). Effect of corporal punishment on students’ motivation and classroom learning. Rev. Eur. Stud. 5:130. doi: 10.5539/res.v5n4p130, 38158624

[ref2] Anderson K. P. Ritter G. W. Zamarro G. (2019). Understanding a vicious cycle: the relationship between student discipline and student academic outcomes. Educ. Res. 48, 251–262. doi: 10.3102/0013189x19848720

[ref3] Astin A. W. (1974). Measuring the outcomes of higher education. New Drctns Instit Rsrch 1974, 23–46. doi: 10.1002/ir.37019740105

[ref4] Baker-Henningham H. Meeks-Gardner J. Chang S. Walker S. (2009). Experiences of violence and deficits in academic achievement among urban primary school children in Jamaica. Child Abuse Negl. 33, 296–306. doi: 10.1016/j.chiabu.2008.05.011, 19481803

[ref5] Davison C. B. Dustova G. (2017). A quantitative assessment of student performance and examination format. J. Instruct. Pedagog. Available online at: https://www.semanticscholar.org/paper/A-Quantitative-Assessment-of-Student-Performance-Davison-Dustova/3e639462cc33767be25f32ffa1be4037fd410fa2 (Accessed July 24, 2025).

[ref6] Devries K. M. Child J. C. Allen E. Walakira E. Parkes J. Naker D. (2014). School violence, mental health, and educational performance in Uganda. Pediatrics 133, e129–e137. doi: 10.1542/peds.2013-2007, 24298004

[ref7] Duxbury S. W. Haynie D. L. (2020). School suspension and social selection: labeling, network change, and adolescent, academic achievement. Soc. Sci. Res. 85:102365. doi: 10.1016/j.ssresearch.2019.102365, 31789197

[ref8] Ferguson C. J. (2013). Spanking, corporal punishment and negative long-term outcomes: A meta-analytic review of longitudinal studies. Clin. Psychol. Rev. 33, 196–208. doi: 10.1016/j.cpr.2012.11.002, 23274727

[ref9] Gershoff E. T. (2017). School corporal punishment in global perspective: prevalence, outcomes, and efforts at intervention. Psychol. Health Med. 22, 224–239. doi: 10.1080/13548506.2016.1271955, 28064515 PMC5560991

[ref10] Gohanna S. J. (2017). Examining the Relationship among Reading Coaches, Student Achievement, and Accommodation Status of Third Grade Students Taking the ACT Aspire Reading Assessments—ProQuest. Available online at: https://www.proquest.com/openview/5c06ec7563df694929f8a573e6754916/1?pq-origsite=gscholar&cbl=18750 (Accessed July 24, 2025).

[ref11] Gregory A. Allen J. P. Mikami A. Y. Hafen C. A. Pianta R. (2014). Eliminating the racial disparity in classroom exclusionary discipline. J. Appl. Res. Child 5, 1–22. doi: 10.58464/2155-5834.1212, 41439502

[ref12] Hall L. Bennett J. Craighead K. (2022). An Examination of Data Meetings in a Private Christian School: Achievement Outcomes and Predictive Relationship between Benchmarks and ACT Aspire Summative Assessment—ProQuest. Available online at: https://www.proquest.com/openview/e4b9d8765db10a29b2b4eaba532caf6d/1?pq-origsite=gscholar&cbl=18750&diss=y (Accessed July 24, 2025).

[ref13] Herbart J. F. (2017). Allgemeine Pädagogik: Aus dem Zwecke der Erziehung Abgeleitet. Forgotten Books. Available online at: https://www.amazon.com/-/zh/dp/0331582929/ref=monarch_sidesheet_title (Accessed June 15, 2025).

[ref14] Hwang N. (2018). Suspensions and achievement: varying links by type, frequency, and subgroup. Educ. Res. 47, 363–374. doi: 10.3102/0013189x18779579

[ref15] Ibrahim H. Johnson O. (2020). School discipline, race–gender and STEM readiness: A hierarchical analysis of the impact of school discipline on math achievement in high school. Urban Rev. 52, 75–99. doi: 10.1007/s11256-019-00513-6

[ref16] Jabbari J. Johnson O. (2020). Veering off track in U.S. high schools? Redirecting student trajectories by disrupting punishment and math course-taking tracks. Child Youth Serv. Rev. 109:104734. doi: 10.1016/j.childyouth.2019.104734

[ref17] Jabbari J. Johnson O. (2023). The collateral damage of in-school suspensions: a counterfactual analysis of high-suspension schools, math achievement and college attendance. Urban Educ. 58, 801–837. doi: 10.1177/0042085920902256

[ref18] Jordan J. L. Anil B. (2009). Race, gender, school discipline, and human capital effects. J. Agric. Appl. Econ. 41, 419–429. doi: 10.1017/s1074070800002893

[ref19] Keatinge M. W. (1967). The Great Didactic of John Amos Comenius. The Great Didactic of John Amos Comenius. Available online at: https://ursecure.roehampton.ac.uk/digital-collection/froebel-archive/great-didactic/index.html (Accessed July 24, 2025).

[ref20] Kim J. (2021). Gender differences in the educational penalty of delinquent behavior: evidence from an analysis of siblings. J. Quant. Criminol. 37, 179–216. doi: 10.1007/s10940-020-09450-0

[ref21] Kinsler J. (2009). Suspending the Right to an Education or Preserving It? An Equilibrium Model of Student Behavior, Achievement, and Suspension. Toronto: Canadian Center of Science and Education.

[ref22] Kodelja Z. (2019). Violence in schools: zero tolerance policies. Ethics Educ. 14, 247–257. doi: 10.1080/17449642.2019.1587682

[ref23] Kralevich M. Slate J. R. Tejeda-Delgado C. Kelsey C. (2010). Disciplinary Methods and Student Achievement: a Statewide Study of Middle School Students. Available online at: http://cnx.org/content/m33878/1.1/

[ref24] Lacoe J. Steinberg M. P. (2018). Rolling back zero tolerance: the effect of discipline policy reform on suspension usage and student outcomes. Peabody J. Educ. 93, 207–227. doi: 10.1080/0161956x.2018.1435047

[ref25] Lehmann P. S. Meldrum R. C. (2021). School suspension in Florida: the interactive effects of race, ethnicity, gender, and academic achievement. Justice Q. 38, 479–512. doi: 10.1080/07418825.2019.1688853

[ref26] Lockex J. Quick R. H. (1880). Some Thoughts Concerning Education Library of Congress Washington, D.C. 20540 USA. Available online at: https://www.loc.gov/resource/gdcmassbookdig.somethoughtsconc00lock/?sp=3&st=gallery

[ref27] Makaranko A. S. Litvinov I. Litvinov T. (1955). The road to life: an epic of Education. 2nd ed. Foreigh Language Publishing House. Available online at: https://cir.nii.ac.jp/crid/1130000794312093824 (Accessed July 24, 2025).

[ref28] Morris E. W. Perry B. L. (2016). The punishment gap: school suspension and racial disparities in achievement. Soc. Probl. 63, 68–86. doi: 10.1093/socpro/spv026

[ref29] Nelson J. (2020). The Impact of Suspension on the Academic Performance of Middle School Students. Theses and Dissertations from 2020. Available online at: https://orc.library.atu.edu/etds_2020/5 (Accessed July 1, 2025).

[ref30] Oplatka I. Tubin D. (2009). The weaknesses and shortcomings of the junior high school in Israel: some insights into grade configurations of educational systems. Int. J. Educ. Reform 18, 200–223. doi: 10.1177/105678790901800303

[ref31] Peguero A. A. Popp A. M. Shekarkhar Z. (2015). Breaking stereotypes and school punishment: family socioeconomic status, test scores, academic and sport activities, backlash, and racial and ethnic discipline disparities. J. Ethn. Crim. Justice 13, 59–85. doi: 10.1080/15377938.2014.893219

[ref32] Peguero A. A. Shekarkhar Z. (2011). Latino/a student misbehavior and school punishment. Hisp. J. Behav. Sci. 33, 54–70. doi: 10.1177/0739986310388021

[ref33] Perry B. L. Morris E. W. (2014). Suspending progress. Am. Sociol. Rev. 79, 1067–1087. doi: 10.1177/0003122414556308

[ref34] Raffaele Mendez L. M. (2003). Predictors of suspension and negative school outcomes: A longitudinal investigation. New Dir. Youth Dev. 2003, 17–33. doi: 10.1002/yd.52, 14635432

[ref35] Rodriguez L. A. Welsh R. O. (2022). The dimensions of school discipline: toward a comprehensive framework for measuring discipline patterns and outcomes in schools. AERA Open 8. doi: 10.1177/23328584221083669

[ref36] Skiba R. J. Chung C.-G. Trachok M. Baker T. L. Sheya A. Hughes R. L. (2014). Parsing disciplinary disproportionality: contributions of infraction, student, and school characteristics to out-of-school suspension and expulsion. Am. Educ. Res. J. 51, 640–670. doi: 10.3102/0002831214541670

[ref37] Smith D. (2015). The Diverse Nature of Corporal Punishment: An Investigation of the Relationship Between Students’ Perceptions of the Discipline Method, Academic Performance, and Social Behaviors. Electronic Theses and Dissertations. Available online at: https://egrove.olemiss.edu/etd/510 (Accessed July 1, 2025).

[ref38] Straus M. A. (2001). New evidence for the benefits of never spanking. Soc. Sci. Public Policy 38, 52–60. doi: 10.1007/BF02712591, 30311153

[ref39] Sullivan A. L. Klingbeil D. A. Van Norman E. R. (2013). Beyond behavior: multilevel analysis of the influence of sociodemographics and school characteristics on students’ risk of suspension. Sch. Psychol. Rev. 42, 99–114. doi: 10.1080/02796015.2013.12087493

[ref40] Tarkington K. A. (2025). Impact of One-to-One Technology on High School Students’ Act Aspire Reading Scores—ProQuest. Available online at: https://www.proquest.com/openview/6f5cf4aa6a13ec7faf1a0eca6c9522e7/1?pq-origsite=gscholar&cbl=18750&diss=y (Accessed July 24, 2025).

[ref41] Umeh Z. Bumpus J. P. Harris A. L. (2020). The impact of suspension on participation in school-based extracurricular activities and out-of-school community service. Soc. Sci. Res. 85:102354. doi: 10.1016/j.ssresearch.2019.102354, 31789193

[ref42] Visser L. N. van der Put C. E. Assink M. (2022). The association between school corporal punishment and child developmental outcomes: a meta-analytic review. Children 9:383. doi: 10.3390/children9030383, 35327755 PMC8946887

[ref43] Welsh R. O. (2022). Overlooked exclusionary discipline: examining placement in alternative schools, expulsions, and referrals to hearing in an urban district. Educ. Policy 36, 550–586. doi: 10.1177/0895904820901481

